# Autophagy Inhibitor (LY294002) and 5-fluorouracil (5-FU) Combination-Based Nanoliposome for Enhanced Efficacy Against Esophageal Squamous Cell Carcinoma

**DOI:** 10.1186/s11671-018-2716-x

**Published:** 2018-10-17

**Authors:** Ye Feng, Yongjian Gao, Dayu Wang, Zhonghang Xu, Weixuan Sun, Ping Ren

**Affiliations:** 1 Department of Gastrointestinal Surgery, China-Japan Friendship Hospital of Jilin University, Changchun, Jilin, 130033 China; 2 Department of Thoracic Surgery, The First Hospital of Jilin University, Changchun, Jilin, 130033 China

**Keywords:** Esophageal cancer, 5-fluorouracil, Autophagy inhibitor, Apoptosis, Liposome

## Abstract

In this study, 5-fluorouracil (5-FU) and LY294002 (LY)-loaded PEGylated nanoliposome was prepared to target esophageal squamous cell carcinoma (ESCC). The particles were characterized in terms of physicochemical and biological parameters. The co-delivery of autophagy inhibitor and chemotherapeutic drug in a single carrier was successfully accomplished. The two components from 5-FU and LY-loaded PEGylated nanoliposome (FLNP) released in a controlled manner with LY relatively released faster compared to that of 5-FU. FLNP showed a receptor-mediated cellular uptake that will allow the gradual release of drug in the acidic environment. The cellular uptake of nanoparticles (NP) was further confirmed by FACS analysis. The combination of 5-FU and LY resulted in higher cytotoxic effect compared to that of individual drugs. Most importantly, FLNP exhibited a significantly higher anticancer effect in cancer cells compared to that of free cocktail combinations. The faster release of LY from FLNP leads to autophagy inhibition that improves the sensitivity of cancer cells towards 5-FU, resulting in more cell death. Consistently, FLNP induced a greater apoptosis (~ 48%) of cancer cells compared to that of any other groups. Western blot analysis clearly showed that 5-FU and LY individually increased the expression of caspase-3 and PARP, while as expected FLNP induced a remarkable expression of these protein markers indicating the superior anticancer effect. We believe that the programmed release of autophagy inhibitor and chemotherapeutic drug from a single nanocarrier will increase the prospect of anticancer therapy in ESCC.

## Background

Esophageal squamous cell carcinoma (ESCC) is one of the common types of esophageal cancers which are more prevalent in East Asia like China [1, 2]. The 5-year survival rate of ESCC is around 25%. Surgery is the main treatment option for ESCC treatment; however, it is believed that adjuvant chemotherapy will have a great impact on the ESCC treatment [3, 4]. It has been reported that chemotherapeutic treatment based on a single drug regimen resulted in poor therapeutic efficacy owing to the heterogeneity of cancer cells [5]. Cancer cells are often resistant to single anticancer drugs and will not be effective in nature. In this regard, a combination of two or more drugs will act in a synergistic manner by targeting different cellular targets [6, 7]. The autophagy inhibitors have been reported to overcome multidrug resistance (MDR) of cancer cells. When cells have limited nutrition and growth factor, autophagy provides the much-needed homeostasis to the cancer environment and contributes for its survival [8]. Autophagy also serves to protect the cancer cells from chemotherapeutic agents. In this study, we have selected LY294002 (LY) as an autophagy inhibitor [9].

Several studies have reported that autophagy inhibitors work best when combined with an anticancer drug. In this study, we have selected 5-fluorouracil (5-FU) as a chemotherapeutic agent. 5-FU has been mainly indicated in the treatment of squamous cell cancers and used in patients with breast and other cancers [10]. 5-FU has a fluorine atom in the C-5 position of the uracil in the place of hydrogen. The anticancer effect of 5-FU arise from the regulation of various molecules such as Bax and Bcl-2 and induce cancer cell apoptosis [11, 12]. Despite the potential cytotoxic effect of 5-FU and LY, both the agents suffer from poor water solubility and stability concerns which results in short plasma half-life and undesirable toxic effects to the normal cells [6]. Moreover, a simple cocktail mixture of both the anticancer agents does not induce a synergistic effect due to the random release of drugs and uncontrolled distribution of drugs in various tissues [7]. Therefore, it is utmost important to deliver both the anticancer drugs in a programmed and much-controlled manner which will increase its therapeutic efficacy.

Nanoparticles have been widely investigated as drug delivery carriers for cancer-targeting applications [13]. The poorly soluble drug could be stably incorporated in the nanoparticles and thereby its solubility and bioavailability could be improved. Among all the carriers, liposome has been widely studied for its suitability for systemic applications [14, 15]. Previous studies have demonstrated that many commercial liposomal formulations are available including Doxil, Myocet, LipoPlatin and so on. The systemic circulation of liposome could be improved by the surface conjugation of polyethylene (glycol) (PEG) as a hydrophilic shell [16]. The nanosize and hydrophilic layer of PEG would confer the prolonged blood circulation and reduce the reticuloendothelial (RES)-mediated plasma clearances. Besides, NP could passively accumulate in the tumor tissues via enhanced permeation and retention (EPR) effect [17, 18]. Therefore, it can be expected that if the chemotherapeutic drug and autophagy inhibitor could be incorporated in the single carrier, it will have a synergistic anticancer effect in the ESCC.

Thus far, the main aim of the present study was to administer a combination of 5-FU and LY to treat ESCC. For this purpose, 5-FU and LY were stably incorporated in the PEGylated nanoliposome. It has been expected that the early release of autophagy inhibitor (LY) will disrupt the protective mechanism of cancer cells and thereafter 5-FU will act in a stronger manner in the cancer cells. The drug-loaded liposome was characterized in terms of particle size, shape and release pattern. Cellular uptake and cell viability assay have been performed in ESCC cancer cells. Furthermore, apoptosis assay was performed by means of annexin V/PI staining and Hoechst 33382 staining. Finally, antitumor efficacy study was performed in ESCC cell-bearing xenograft animal model.

## Methods

### Materials

5-Fluorouracil was purchased from Sigma-Aldrich, China. 2-(4-morpholinyl)-8-phenyl-4H-1-benzopyran-4-one (LY294002, LY) was purchased from Beijing Huafeng United Technology Corporation, China. L-a-phosphatidylcholine (egg/chicken) (EPC), disteroylphosphatidylethanolamine-polyethylene glycol (2000) (DSPE-PEG) and cholesterol were purchased from Avanti Polar Lipids, China. All other chemicals were of reagent grade and used for further purifications.

### Preparation of Dual Drug-loaded Nanoliposomes

EPC, DSPE-PEG and cholesterol were mixed in a methanol-chloroform mixture (10:4:1 M ratio (total 10 mg)), and to this organic mixture, 5-FU (1.5 mg) and LY (1.5 mg) were added. The organic solvents were evaporated using a rotary evaporator (Buchi, USA) at 60 °C for 1 h. The thin lipid film was hydrated with the PBS buffer for 1 h and followed by extruded through a mini-extruder via a polycarbonate membrane (200 nm). The drug-loaded nanoliposome was filtered through a Millipore tube with an MW of 3500. The drug-loaded liposome was collected from the upper chamber and the unentrapped drug was determined by the HPLC method. The drug loading and encapsulation were calculated using the following equations:

$$ \mathrm{DL}\ \left(\%\right)=\left(\mathrm{Weight}\ \mathrm{of}\ \mathrm{loaded}\ \mathrm{drug}/\mathrm{weight}\ \mathrm{of}\ \mathrm{liposome}\right)\times 100\% $$

$$ \mathrm{EE}\ \left(\%\right)=\left(\mathrm{Weight}\ \mathrm{of}\ \mathrm{loaded}\ \mathrm{drug}/\mathrm{weight}\ \mathrm{of}\ \mathrm{drug}\ \mathrm{initially}\ \mathrm{added}\right)\times 100\% $$

### Particle Size Analysis

The particle size and size distribution of nanoparticle were determined by dynamic light scattering (DLS) technique using Zetasizer (Nano ZS, Malvern Instruments, UK). The particle size was measured at 25 °C after diluting appropriately with distilled water. All measurements were performed in triplicate.

### Morphology Analysis

The morphology of NP was first studied by transmission electron microscopy (TEM; H-7600, Hitachi, Tokyo, Japan) at an accelerating voltage of 100 kV. The samples were loaded in a carbon-coated copper grid and dried. The NP morphology was further observed in atomic force microscopy (AFM). The samples were placed on a mica surface.

### In Vitro Release Study

The in vitro release of 5-FU and LY from 5-FU and LY-loaded PEGylated nanoliposome (FLNP) was studied in phosphate buffered saline (PBS, pH 7.4) at 37 °C. The NP dispersion (1 ml containing 1 mg of each drug in a carrier) was placed in a dialysis membrane (MW ~ 3500) and both the ends were sealed. The dialysis membrane was placed in a tube with 30 ml of release medium. The tube containing dialysis membrane was placed in a shaker bath and allowed to incubate for prerequisite time point. At specific time points, 100 μl of the sample was withdrawn and replaced with an equal amount of fresh buffer. The amount of drug released in the buffer was studied by means of the HPLC method. A high-performance liquid chromatography (HPLC) system (Hitachi, Tokyo, Japan) that consist of L-2200 autosampler and L-2420 UV–Vis detector was used. An Inertsil C18 Column (150 mm × 4.6 mm, 5 μm particle size; Cosmosil, Nacalai Tesque Inc., USA) was used under isocratic elution of the mobile phase at a flow rate of 1.0 mL/min and a column temperature of 25 °C. Good linearity was observed between 0.025 and 2 μg/ml for both drugs with a correlation coefficient (*r*^2^) around 0.9995. The LOD (μg/ml) for 5-FU and LY were 0.020 and 0.050, respectively. The LOQ (μg/ml) for 5-FU and LY were 0.070 and 0.150, respectively.

### Cell Culture

The esophageal cancer cell line, EC 9706, was cultured in normal RPMI media with 10% FBS and 100 units/mL penicillin, 100 mg/ml streptomycin in 5% CO_2_ and 95% humidity atmosphere in the humidifier.

### Cellular Uptake Analysis

The cellular uptake of NP was observed by confocal laser scanning microscope (CLSM). For this purpose, rhodamine B-loaded NP was prepared and purified by gel filtration using Sephadex G-50 column with HEPES buffer. The cells were seeded in 12-well plates and incubated overnight. The cells were then exposed with rhodamine B-loaded NP (10 μg/ml) and incubated for 2 h. The cells were washed with PBS three times and fixed with 2% paraformaldehyde (PFA) and washed again with PBS. The cells were then stained with DAPI as a nuclear staining agent. The cells were washed and observed under a fluorescence microscope (Nikon TE2000-E microscope).

The cellular uptake was further observed using flow cytometer. The cells were seeded in 12-well plates and incubated overnight. The cells were then exposed with rhodamine B-loaded NP and incubated for 1 h and 2 h, respectively. The cells were then extracted and washed twice with PBS. The cells were resuspended in PBS buffer and analyzed using a Calibur fluorescence-activated cell sorter equipped with CELLQuest software (Becton Dickinson Biosciences, San Jose, CA, USA).

### Cytotoxicity Assay

The cytotoxic potential of individual formulations was evaluated by 3-(4,5-dimethyl-2-thiazoyl)-2,5-diphenyl tetrazolium bromide (MTT) assay. Briefly, 1 × 10^4^ cells were seeded in a 96-well plate and incubated for 24 h. The cells were then exposed with different concentrations of free 5-FU, LY, 5-FU + LY and FLNP, respectively and further incubated for 24 h. MTT solutions were prepared (5 mg/ml), and 10 μl of MTT solution was added to each well and incubated for 4 h. The formazan crystal was extracted by adding DMSO, and the absorbance was studied using a microplate reader (Synergy™ HTX Multi-Mode Microplate Reader) (at 570 nm).

### Apoptosis Assay

The apoptosis of the cancer cell was determined by Annexin-V/PI staining protocol (BD Biosciences, USA). Briefly, EC 9706 was seeded in a 12-well plate and incubated for 24 h. The cells were incubated with free 5-FU, LY, 5-FU + LY and FLNP, respectively and further incubated for 24 h (1 μg of equivalent drug concentration). The cells were extracted and stained with annexin V-FITC and PI for 15 min at room temperature and then enumerated via flow cytometry analysis using a FACS CELLQuest software (Becton Dickinson Biosciences, San Jose, CA, USA).

### Hoechst 33342 Assay

The apoptosis of the cancer cell was further determined by Hoechst 33342 staining protocol. Briefly, EC 9706 was seeded in a 12-well plate and incubated for 24 h. The cells were incubated with free 5-FU, LY, 5-FU + LY and FLNP, respectively and further incubated for 24 h (1 μg of equivalent drug concentration). The cells were fixed with 2% PFA and incubated with Hoechst 33342 (1 μg/ml) for 15 min. The cell apoptosis was then observed under a fluorescence microscope.

### Western Blotting

EC 9706 cells were seeded and treated with free 5-FU, LY, 5-FU + LY and FLNP, respectively and further incubated for 24 h. The cells were then lysed and the supernatant was subjected to sodium dodecyl sulfate–polyacrylamide gel electrophoresis (SDS-PAGE) and blotted onto polyvinylidene difluoride membrane (Millipore). Membranes were blocked with 5% skim milk and subsequently incubated with the specific primary antibodies overnight at 4 °C. After incubation with the secondary antibody conjugate horseradish peroxidase, blots were revealed using the ECL system (AbClon) for signal detection. Films were developed using a Kodak M35-A X-OMAT processor.

### In Vivo Tumour Growth Inhibition

The animal studies were performed as per the guidelines famed by the Institutional Animal Care and Use Committee, Subei People’s Hospital of Jiangsu Province, China. A 6-week old female nude mice were inoculated with 1 × 10^6^ OE-19 cells on the right flank of the mice in a 100 μl of media. The tumours were allowed to grow until 80 mm^3^ (day 10). The animal was divided into five groups with six mice in each group. The mice were injected with respective formulations at a fixed dose of 5 mg/kg and administered three times during the first 10 days of the experiment. The tumor size was measured in terms of tumor volume using vernier calipers and the size estimated using the formula; Volume = (width^2^ × length)/2.

### Statistical Analysis

Data were expressed as mean ± standard deviations. The statistical significance was determined using *t* test and analysis of variance (ANOVA). *P* < 0.05 was considered statistically significant.

## Results and Discussion

**Fig. 1:**
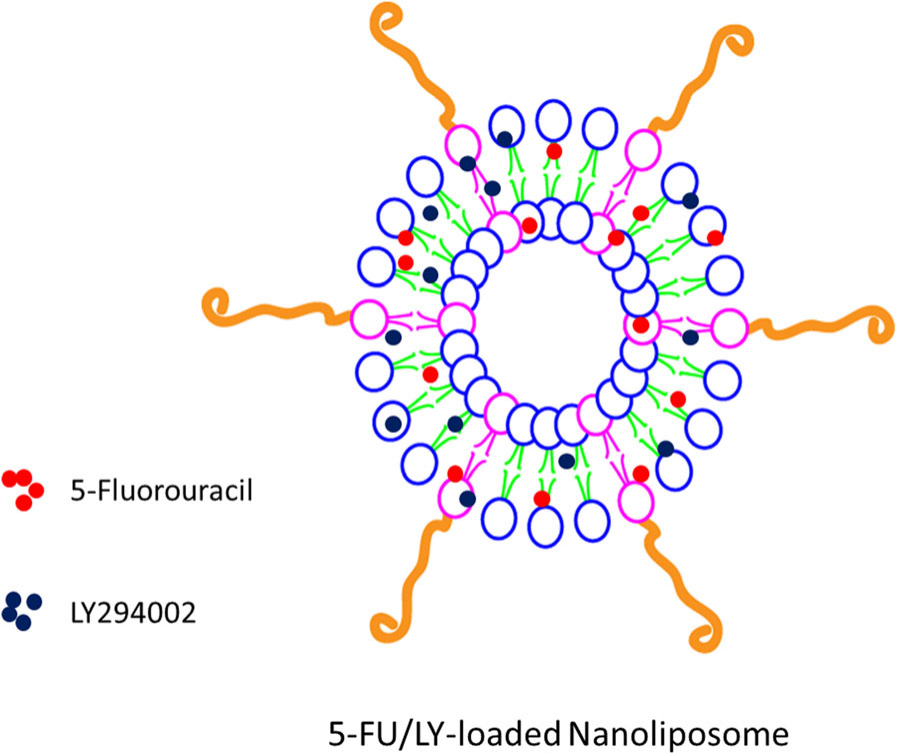
Schematic illustration of the preparation of 5-fluorouracil and LY-loaded PEGylated nanoliposomes

Cancer cells are often resistant to single anticancer drugs and will not be effective in nature. In this regard, a combination of two or more drugs will act in a synergistic manner by targeting different cellular targets. The autophagy inhibitors have been reported to overcome multidrug resistance (MDR) of cancer cells. Several studies have reported that autophagy inhibitors work best when combined with an anticancer drug. In this study, we have selected 5-fluorouracil (5-FU) as a chemotherapeutic agent and LY294002 (LY) as an autophagy inhibitor. In order to address the solubility and stability concerns of free drugs which in turn results in short plasma half-life and undesirable toxic effect, we have stably incorporated 5-FU and LY in the PEGylated nanoliposome (Fig. 1).

### Preparation and Characterization of 5-FU and LY-loaded Nanoliposomes

**Fig. 2:**
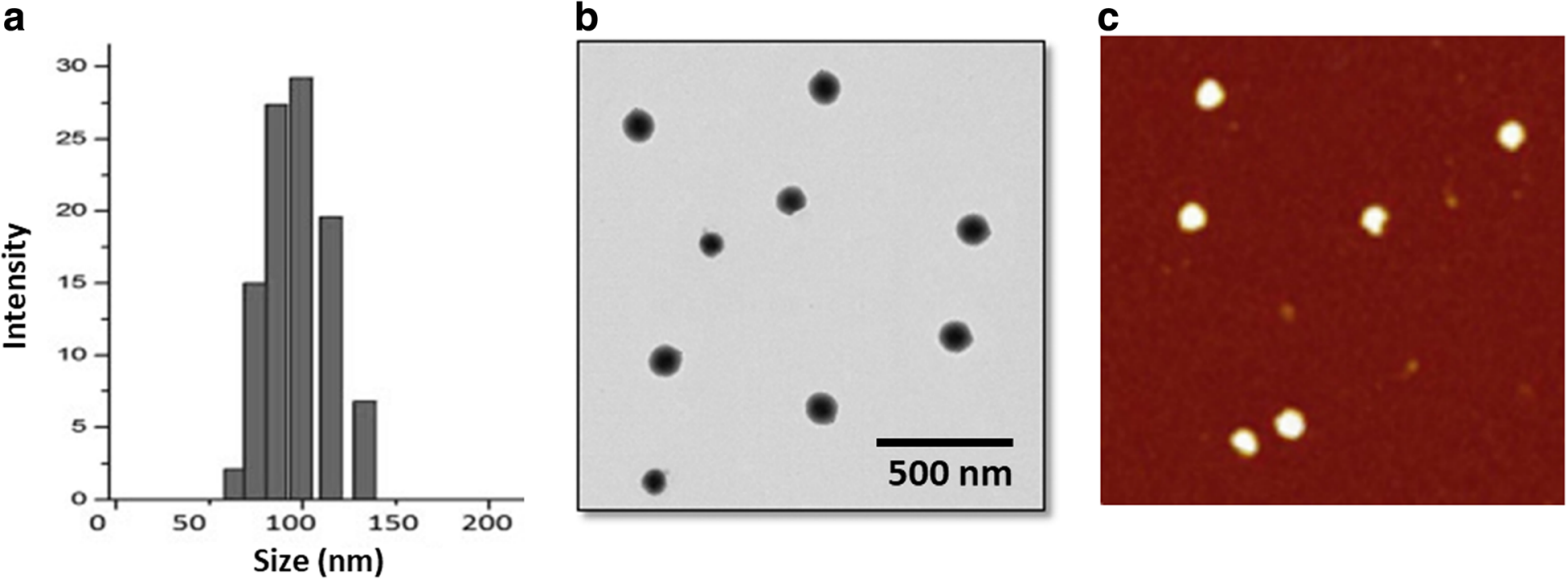
Physicochemical characterizations of FLNP. **a** Particle size distribution of FLNP by DLS method. **b** TEM image of FLNP. **c** AFM image of FLNP

The dual-drug loaded liposome was prepared by thin-film hydration method. The average particle size of blank liposome was found to be ~ 110 nm with an excellent dispersity index. The particle size of dual drug-loaded liposome (FLNP) increased to ~ 150 nm with a good polydispersity index (PDI ~ 150) (Fig. 2a). The increase in particle size was mainly attributed to the incorporation of hydrophobic drugs in the liposome. It has been reported that particle size around 200 nm will enhance the accumulation of drug in the tumor tissues by virtue of enhanced permeation and retention effect (EPR). The drug-loaded liposome exhibited a long-term storage ability attributed to the presence of PEG on its surface that contributed to the anti-fouling effect. Moreover, FLNP exhibited a mean surface charge of ~ 25 mV indicating its colloidal stability. The negative surface charge will avoid any interactions in the systemic circulations.

The morphology of FLNP was first observed using TEM (Fig. 2b). As seen, particles were typical spherical shaped and uniformly dispersed in the copper grid. A slight greyish shell on the circumference was attributed to the presence of PEG on its surface. The particle size observed from TEM was slightly smaller compared to that from DLS. The particle size from TEM was ~ 130 nm compared to ~ 150 nm from DLS. The difference in size of particles from two techniques might be attributed to the state of measurement. The TEM measures the particle in dried conditions while DLS measures the particle in hydrated conditions and the PEG chain elongation. The particle morphology was further confirmed by AFM (Fig. 2c). The particles were circular and present as a flattened object on the mica surface.

### Drug Loading and In Vitro Drug Release

**Fig. 3:**
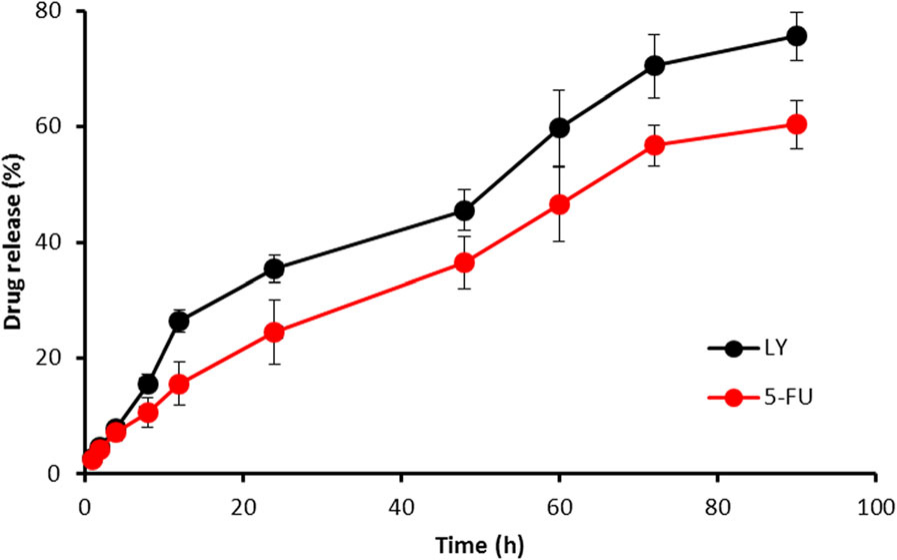
Release profiles of 5-FU and LY from FLNP in phosphate-buffered saline (PBS, pH 7.4). The amount of drug released was quantified by HPLC analysis

FLNP showed a high entrapment efficiency for both the drugs (92.5% for 5-FU and 86% for LY) with an active drug loading of about ~ 16% *w*/*w*. The in vitro release behavior of 5-FU and LY from FLNP was observed in phosphate-buffered saline (pH 7.4). The two components from FLNP released in a controlled manner in pH 7.4 conditions. For example, ~ 30% of 5-FU and ~ 40% of LY released from the liposome by the end of 24 h. The release of drugs continued until 90 h with ~ 60% of 5-FU and ~ 75% of LV released (Fig. 3). It should be noted that the release rate significantly decreased after 24 h until 90 h indicating the slow diffusion of drugs from the NP system. Such controlled release of the drug would be beneficial for the cancer-targeting applications. Furthermore, a slow release of drug in the neutral conditions indicates lesser side effects to the normal tissues. It is worth noting that LY relatively released faster compared to that of 5-FU. It will be a beneficial situation as the LY would make the cancer cells more sensitive to 5-FU resulting in enhanced cytotoxic effect in the tumor tissues.

### Cellular Internalization Study

**Fig. 4:**
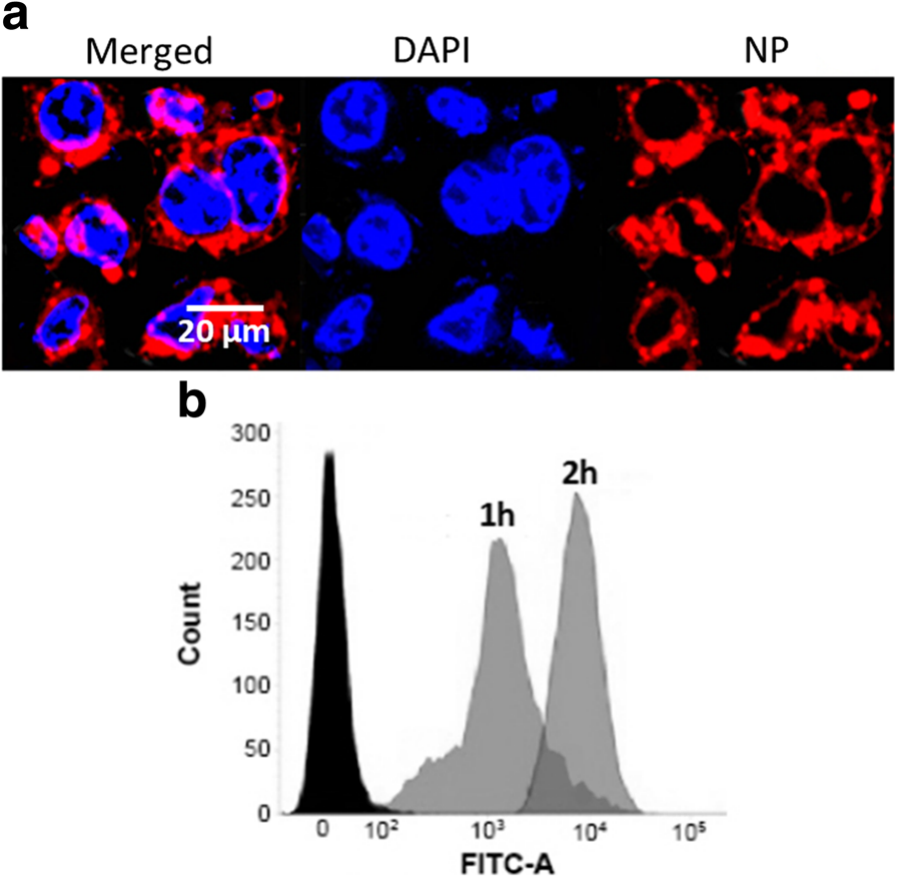
**a** Confocal laser scanning microscope (CLSM) image of HT-29 cancer cells after incubation with FLNP for 2 h. The nucleus was stained with DAPI and Rhodamine B was used as a fluorescent probe. **b** Flow cytometer analysis of FLNP upon incubation for different time points

Confocal laser scanning microscopy (CLSM) was performed to determine the subcellular pattern of FLNP. The rhodamine B was used as a fluorescent probe to track the intracellular uptake of the NPs in ESCC. As seen (Fig. 4), a strong red fluorescence signal was observed in the cellular cytoplasm on the circumference of the nucleus indicating the typical endocytosis-mediated cellular uptake. The receptor-mediated cellular uptake will allow the gradual release of drug in the acidic environment. The nanosize of particle allowed the easy internalization of NP system. The results clearly showed that drug entered the cancer cells via a specific pathway rather than the simple diffusion. The cellular uptake was further monitored by FACS. As seen, remarkably uptake of NP was observed after 1 h incubation and the cellular uptake increased with the increase in the incubation time (2 h). The greater cellular uptake of NP will increase the intracellular concentration of encapsulated anticancer drugs that will further increase the therapeutic efficacy in ESCC.

### In Vitro Anticancer Effect

**Fig. 5:**
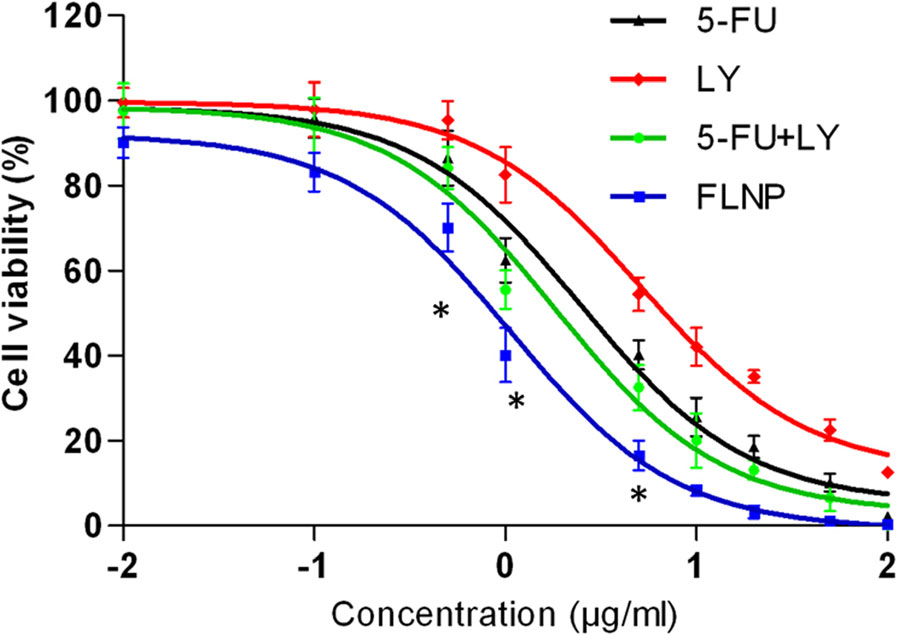
Cell viability of HT-29 cancer cells upon incubation with free 5-FU, LY, 5-FU + LY and FLNP, respectively. The cell viability was determined by MTT assay

The cytotoxic effect of individual formulations was studied by MTT assay (Fig. 5). As shown, free drugs as well as combinational NP exhibited a typical concentration-dependent cytotoxic effect in ESCC. The 5-FU exhibited a relatively higher anticancer effect compared to that of LY in the cancer cells. The combination of 5-FU and LY resulted in higher cytotoxic effect compared to that of individual drugs. Most importantly, FLNP exhibited a significantly higher anticancer effect in cancer cells compared to that of free cocktail combinations. The IC50 value of 5-FU, LY, 5-FU + LY and FLNP were stood at 2.68 μg/ml, 5.98 μg/ml, 1.54 μg/ml and 0.58 μg/ml, respectively. This could be attributed to the fact that LY can inhibit the autophagy in the cancer cells and making it more responsive to 5-FU. It should be noted that FLNP was more effective than free cocktail combinations due to a more programmed release of drugs in a controlled manner. The faster release of LY from FLNP leads to autophagy inhibition that improves the sensitivity of cancer cells towards 5-FU, resulting in more cell death [19]. It has been reported that 5-FU after entering the cancer cells converted to fluorouridine triphosphate (FUTP) via fluorouridine monophosphate (FUMP) or metabolized into fluorodeoxyuridine triphosphate (FdUTP) via two different pathways. The FdUTP acts as a substrate to thymidylate synthase (TS) and blocks the nucleotide synthesis. The 5-FU metabolite binds to the nucleotide-binding site of TS and inhibits the nucleotide synthesis. This cycle leads to the depletion of deoxythymidine triphosphate (dTTP) that will prevent the DNA synthesis and results in enhanced cancer cell death [20, 21].

### Apoptosis Essay

**Fig. 6:**
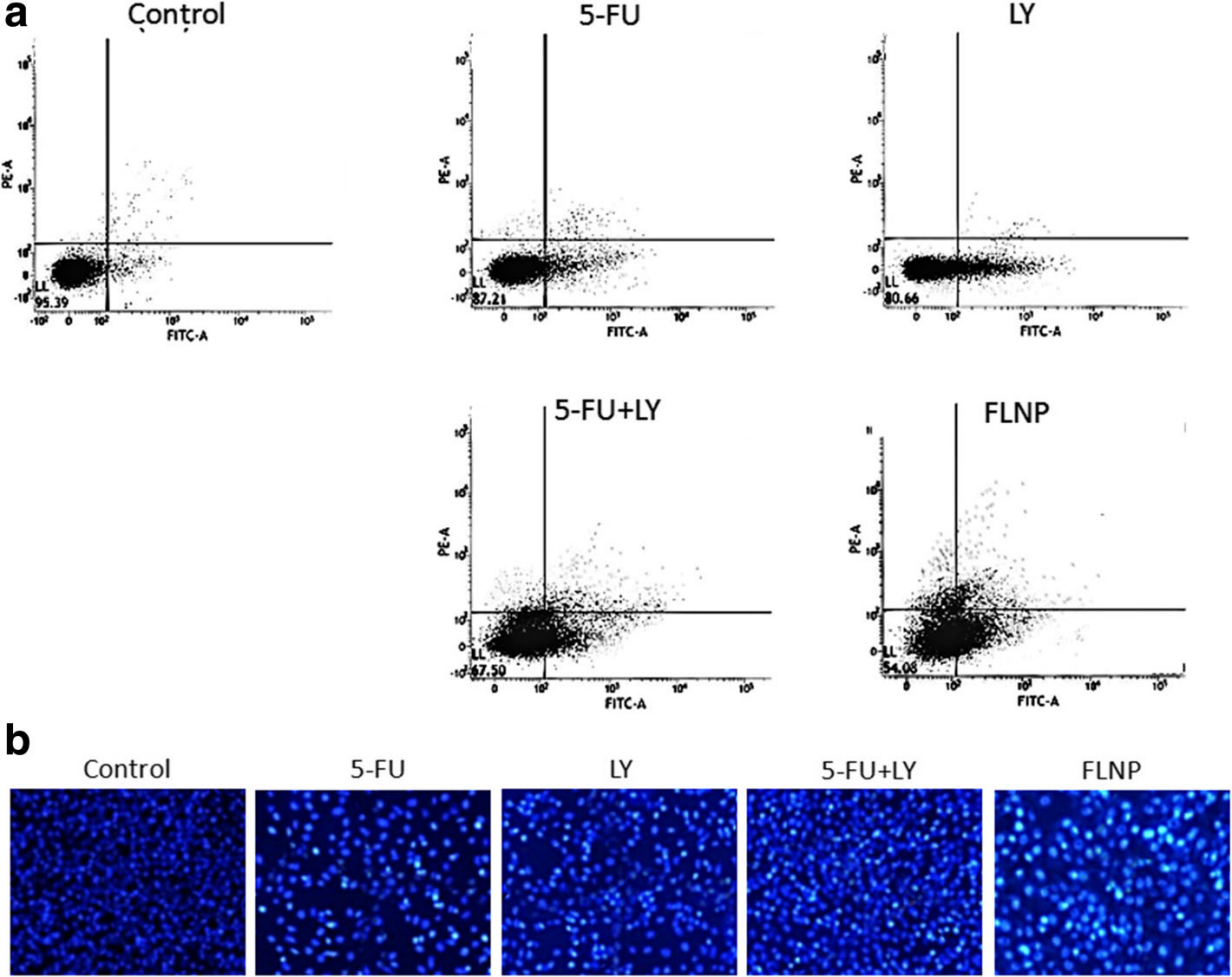
Representative flow cytometric analysis results of cell apoptosis of HT-29 cancer cells upon incubation with free 5-FU, LY, 5-FU + LY and FLNP, respectively. Lower left, living cells; lower right, early apoptotic cells; upper right, late apoptotic cells; and upper left, necrotic cells

The apoptosis effect of the formulation was evaluated by Annexin-V/PI staining method (Fig. 6a, b). This will enable to determine the apoptosis effect of the individual as well as combination drug system. Consistent with the MTT assay, 5-FU (~ 20%) resulted in greater apoptosis of cancer cells compared to that of LY (~ 12%), while 5-FU + LY combination induced greater apoptotic cell death (~ 30%). Compared to any other group, FLNP induced a greater apoptosis (~ 48%) of cancer cells suggesting that receptor-mediated cellular uptake greatly increased the intracellular concentration of anticancer drugs that resulted in higher therapeutic effect. An important reason for the higher anticancer effect of FLNP was mainly attributed to the sequential release of encapsulated drugs wherein LY will sensitize the tumor cells and 5-FU will induce its potent cytotoxic effects. Anticancer therapies work by inducing apoptosis in cancer cells without damaging the surrounding normal cells. The appreciable apoptotic activity of FLNP was mainly attributed to the higher accumulation of nanoparticles in the cells via endocytosis uptake and the sequential release of drugs in the intracellular environment that resulted in a synergistic therapeutic effect. It has been expected that an early release of the autophagy inhibitor (LY) will disrupt the protective mechanism of cancer cells and thereafter 5-FU will act in a stronger manner in the cancer cells.

### Western Blot Analysis

**Fig. 7:**
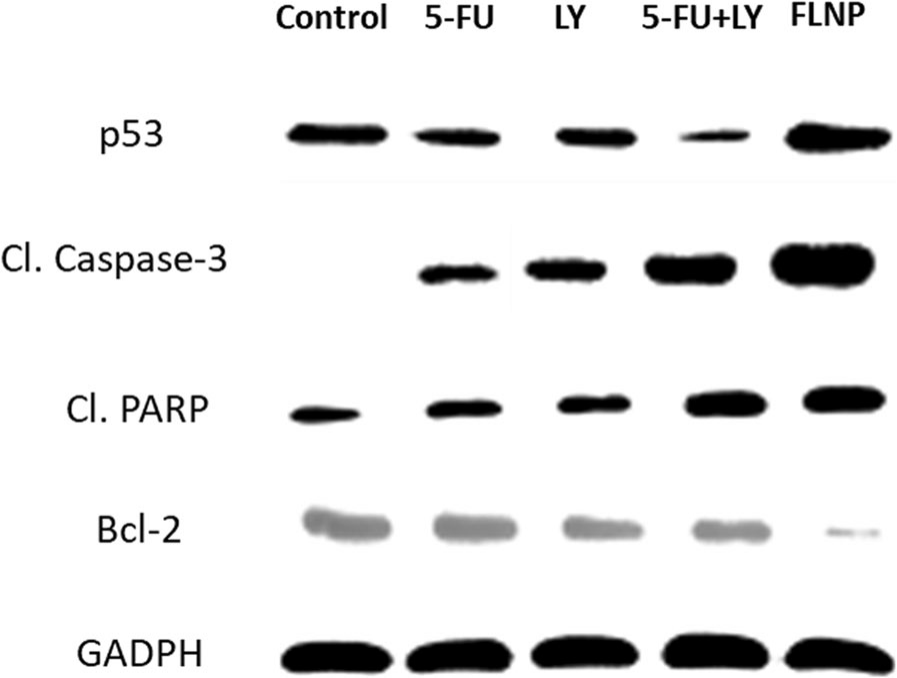
Western blot analysis of HT-29 cancer cells upon incubation with free 5-FU, LY, 5-FU + LY and FLNP, respectively. Western blot analysis of cleaved caspase-3, PARP, Bcl-2 and p53 was determined

**Fig. 8:**
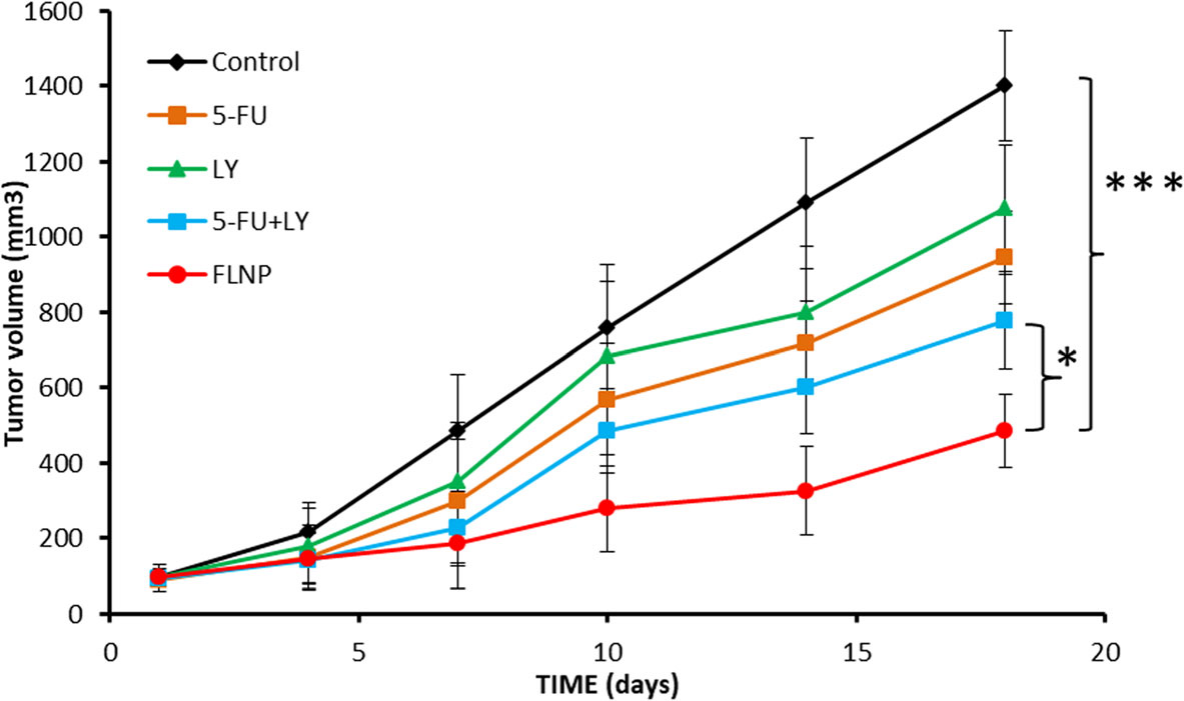
In vivo antitumor efficacy study on animal model. The formulations free 5-FU, LY, 5-FU + LY and FLNP, respectively, were administered to tumor mice, and efficacy was studied for 20 days

The mechanism of action of formulations is determined by Western blot analysis (Fig. 7). The expression of pro-apoptotic and anti-apoptosis proteins levels upon exposure to respective formulations is presented in Fig. 8. p53 is one of the important cell cycle regulator, and its downregulation is associated with the increased survival of cancer cells. It can be seen that formulations significantly downregulated the expression levels of p53 indicating the remarkable effect on the ESCC cells. It has been reported that DNA damage results in the activation of caspase cascade which results in the cleavage of PARP-1 which is considered a hallmark of cell apoptosis [22, 23]. Our results clearly showed that 5-FU and LY individually increased the expression of caspase-3 and PARP, while as expected FLNP induced a remarkable expression of these protein markers indicating the superior anticancer effect. Consistently, FLNP significantly downregulated the expression of the anti-apoptotic protein (Bcl-2), indicating its efficiency to improve the therapeutic efficacy in ESCC.

### In Vivo Tumor Growth Inhibition Analysis

The mice were injected with respective formulations in mice, and the tumor volume was noted until 20 days. As seen (Fig. 8), the control group showed a steady increase in tumor volume with every time point until day 18. Compared to control, free drugs did control the growth of the tumor; however, it was not satisfactory. The cocktail mixture of two drugs was relatively more effective than the individual drugs in controlling the tumor volume. Importantly, FLNP showed significantly (*p* < 0.05; *p* < 0.0001) higher antitumor efficacy compared to any other group. The final tumor volume was ~ 500 mm^3^ compared to ~ 1400 mm^3^ for untreated mice. The significantly higher antitumor effect in tumor model was attributed to the nanosized of particle which might be accumulated in the tumor tissues owing to enhanced permeation and retention effect. The controlled release of drugs in the tumor tissues also contributed to its higher efficacy [24].

## Conclusions

In conclusion, 5-fluorouracil (5-FU) and LY294002 (LY)-loaded PEGylated nanoliposome was successfully prepared to target esophageal squamous cell carcinoma (ESCC). FLNP showed a receptor-mediated cellular uptake that will allow the gradual release of drug in the acidic environment. The combination of 5-FU and LY resulted in higher cytotoxic effect compared to that of individual drugs. Most importantly, FLNP exhibited a significantly higher anticancer effect in cancer cells compared to that of free cocktail combinations. The faster release of LY from FLNP leads to autophagy inhibition that improves the sensitivity of cancer cells towards 5-FU, resulting in more cell death. Consistently, FLNP induced a greater apoptosis (~ 48%) of cancer cells compared to that of any other groups. Western blot analysis clearly showed that 5-FU and LY individually increased the expression of caspase-3 and PARP, while as expected FLNP induced a remarkable expression of these protein markers indicating the superior anticancer effect. We believe that the programmed release of autophagy inhibitor and chemotherapeutic drug from a single nanocarrier will increase the prospect of anticancer therapy in ESCC. A broader study on different squamous cell carcinoma and development of the orthotropic model and patient-derived xenograft (PDX) model will be the subject of future investigation.

## Abbreviations

5-FU: 5-Fluorouracil
EPR: Enhanced permeation and retention effect
ESCC: Esophageal squamous cell carcinoma
FLNP: 5-FU and LY-loaded lipid nanoparticles

## References

[1] Li Q, Tanaka Y, Saitoh Y, Tanaka H, Miwa N (2015). Carcinostatic effects of platinum nanocolloid combined with gamma irradiation on human esophageal squamous cell carcinoma. Life Sci.

[2] Sun LL, Wu JY, Wu ZY, Shen JH, Xu XE, Chen B, Wang SH, Li EM, Xu LY (2015). A three-gene signature and clinical outcome in esophageal squamouscell carcinoma. Int J Cancer.

[3] Luo KJ, Wen J, Xie X, Fu JH, Luo RZ, Wu QL, Hu Y (2012). Prognostic relevance of Id-1 expression in patients with resectable esophageal squamous cell carcinoma. Ann Thorac Surg.

[4] Chen MQ, Xu BH, Zhang YY (2014). Analysis of prognostic factors for esophageal squamous cell carcinoma with distant organ metastasis at initial diagnosis. J Chin Med Assoc.

[5] Wilson TR, Longley DB, Johnston PG (2006). Chemoresistance in solid tumours. Ann Oncol.

[6] Ramasamy T, Ruttala HB, Choi JY, Tran TH, Ku SK, Choi HG, Yong CS, Kim JO (2015). Engineering of a lipid-polymer nanoarchitectural platform for highly effective combination therapy of doxorubicin and irinotecan. Chem Commun.

[7] Ramasamy T, Haidar ZS, Tran TH (2014). Layer-by-layer assembly of liposomal nanoparticles with PEGylated polyelectrolytes enhances systemic delivery of multiple anticancer drugs. Acta Biomater.

[8] Kumar P, Zhang DM, Degenhardt K, Chen ZS (2012). Autophagy and transporter-based multi-drug resistance. Cell.

[9] Han W, Sun J, Feng L, Wang K, Li D, Pan Q, Chen Y, Jin W, Wang X, Pan H, Jin H (2011). Autophagy inhibition enhances daunorubicin-induced apoptosis in K562 cells. PLoS One.

[10] Grem J (2000). 5-fluorouracil: forty-plus and still ticking: a review of its preclinical and clinical development. Investig New Drugs.

[11] Tong D, Poot M, Hu D, Oda D (2000). 5-fluorouracil-induced apoptosis in cultured oral cancer cells. Oral Oncol.

[12] Thant A, Wu Y, Lee J, Mishra D, Garcia H, Koeffler HP (2008). Role of caspases in 5-FU and selenium-induced growth inhibition of colorectal cancer cells. Anticancer Res.

[13] Sundaramoorthy P, Ramasamy T, Mishra SK, Jeong KY, Yong CS, Kim JO, Kim HM (2016). Engineering of caveolae-specific self-micellizing anticancer lipid nanoparticles to enhance the chemotherapeutic efficacy of oxaliplatin in colorectal cancer cells. Acta Biomater.

[14] Allen TM, Cullis PR (2013). Liposomal drug delivery systems: from concept to clinical applications. Adv Drug Deliv Rev.

[15] Sundaramoorthy P, Baskaran R, Mishra SK, Jeong KY, Oh SH, Kyu Yoo B, Kim HM (2015). Novel self-micellizing anticancer lipid nanoparticles induce cell death of colorectal cancer cells. Colloids Surf B Biointerfaces.

[16] Mohan (2016). Dual drug loaded nanoliposomal chemotherapy: a promising strategy for treatment of head and neck squamous cell carcinoma. Eur J Pharm Biopharm.

[17] Ramasamy T, Ruttala HB, Chitrapriya N, Poudal BK, Choi JY, Kim ST, Youn YS, Ku SK, Choi HG, Yong CS, Kim JO (2017). Engineering of cell microenvironment-responsive polypeptide nanovehicle co-encapsulating a synergistic combination of small molecules for effective chemotherapy in solid tumors. Acta Biomater.

[18] Meng L, Yang L, Zhao X, Zhang L, Zhu H, Liu C, Tan W (2012). Targeted delivery of chemotherapy agents using a liver cancer-specific aptamer. PLoS One.

[19] Sheen JH, Zoncu R, Kim D, Sabatini DM (2011). Defective regulation of autophagy upon leucine deprivation reveals a targetable liability of human melanoma cells in vitro and in vivo. Cancer Cell.

[20] Longley D, Harkin D, Johnston P (2003). 5-fluorouracil: mechanisms of action and clinical strategies. Nat Rev Cancer.

[21] Tokunaga E, Oda S, Fukushima M, Maehara Y, Sugimachi K (2000). Differential growth inhibition by 5-fluorouracil in human colorectal carcinomacell lines. Eur J Cancer.

[22] Soldani C, Lazzè MC, Bottone MG (2001). Poly (ADP-ribose) polymerase 807 cleavage during apoptosis: when and where?. Exp Cell Res.

[23] Casiano CA, Ochs RL, Tan EM (1998). Distinct cleavage products of nuclear proteins 810 in apoptosis and necrosis revealed by autoantibody probes. Cell Death Differ.

[24] Ramasamy T, Ruttala HB, Gupta B, Poudel BK, Choi HG, Yong CS, Kim JO (2017). Smart chemistry-based nanosized drug delivery systems for systemic applications: a comprehensive review. J Control Release.

